# Role of the transcription factor Fli-1 on the CXCL10/CXCR3 Axis*

**DOI:** 10.3389/fimmu.2023.1219279

**Published:** 2023-09-15

**Authors:** Xuan Wang, Mara Lennard Richard, Tomika S. Caldwell, Kamala Sundararaj, Shuzo Sato, Tamara K. Nowling, Xian K. Zhang

**Affiliations:** ^1^ Department of General Practice, Xiangya Hospital, Central South University, Changsha, Hunan, China; ^2^ Department of Medicine, Division of Rheumatology & Immunology, Medical University of South Carolina, Charleston, SC, United States; ^3^ Department of Rheumatology, Fukushima Medical University School of Medicine, Fukushima, Japan

**Keywords:** CXCL10, CXCR3, chemokine, friend leukemia virus integration 1 transcription factor, inflammation, lupus nephritis

## Abstract

The transcription factor Fli-1, a member of the ETS family of transcription factors, is implicated in the pathogenesis of lupus disease. Reduced Fli-1 expression in lupus mice leads to decreased renal *Cxcl10* mRNA levels and renal infiltrating CXCR3+ T cells that parallels reduced renal inflammatory cell infiltration and renal damage. Inflammatory chemokine CXCL10 is critical for attracting inflammatory cells expressing the chemokine receptor CXCR3. The CXCL10/CXCR3 axis plays a role in the pathogenesis of various inflammatory diseases including lupus. Our data here demonstrate that renal CXCL10 protein levels are significantly lower in Fli-1 heterozygous MRL/*lpr* mice compared to wild-type MRL/*lpr* mice. Knockdown of Fli-1 significantly reduced CXCL10 secretion in mouse and human endothelial cells, and human mesangial cells, upon LPS or TNFα stimulation. The Fli-1 inhibitor, Camptothecin, significantly reduced CXCL10 production in human monocyte cells upon interferon stimulation. Four putative Ets binding sites in the *Cxcl10* promoter showed significant enrichment for FLI-1; however, FLI-1 did not directly drive transcription from the human or mouse promoters, suggesting FLI-1 may regulate CXCL10 expression indirectly. Our results also suggest that the DNA binding domain of FLI-1 is necessary for regulation of human *hCXCR3* promotor activity in human T cells and interactions with co-activators. Together, these results support a role for FLI-1 in modulating the CXCL10-CXCR3 axis by directly or indirectly regulating the expression of both genes to impact lupus disease development. Signaling pathways or drugs that reduce FLI-1 expression may offer novel approaches to lupus treatment.

## 1 Introduction

Lupus nephritis (LN) is a major cause of mortality in both animal lupus-prone strains and human patients with systemic lupus erythematosus (SLE) (1, 2). Patients with LN have significantly higher mortality compared with patients with non-lupus end stage renal diseases (3, 4). Accumulations of immune complexes and inflammatory cells in kidney are the hallmark of LN (5). At present, the major pathogenesis of LN in renal damage is not fully understood (1). Also, existing data show that current treatments of LN, cytotoxic drugs and corticosteroids, are imperfect with significant side-effects (6, 7). Hence, a better understanding of the complex pathogenesis of LN and identification of novel targets are needed.

Friend leukemia virus integration 1 (Fli-1), a member of the E26 transformation-specific (Ets) transcription factor family, plays a key role in the pathogenesis and development of various diseases including lupus, cancer, systemic sclerosis and sepsis (8–11) (12). In particular, Fli-1 is implicated in the development of lupus prone mouse strains, and elevated expression of Fli-1 is associated with active human lupus nephritis (13–17). Reduced expression of Fli-1 in two lupus mouse strains significantly attenuated renal disease evidenced by decreased infiltrating inflammatory cells and prolonged survival (14, 15). Furthermore, Fli-1 is a key regulator of several inflammatory cytokines and chemokines including monocyte chemotactic protein 1 (MCP-1), chemokine (C-C motif) ligand 5 (CCL5), interleukin 6 (IL-6), granulocyte colony stimulating factor (G-CSF), chemokine ligand 2 (CXCL2) and IL-17A (10, 18–22). Therefore, Fli-1 may affect the pathogenesis of LN as a global modulator of inflammatory mediators.

CXCL9/MIG (monokine induced by interferonγ), CXCL10/IP-10 (interferon inducible 10 protein) and CXCL11/I-TAC (inducible T cell-α chemoattractant), inflammatory chemokines belonging to the CXC chemokine family, and exert their effects by binding to chemokine (C-X-C motif) receptor 3 (CXCR3) (23). These three chemokines lack glutamic acid-leucine-arginine (ELR) and are closely related to each other, with about 40% similarity of amino acid sequence (23). CXCL10 is chemotactic for inflammatory cells including macrophages, monocytes and activated T and NK cells that express CXCR3 (24). Moreover, overexpression of CXCL10 is associated with clinical lupus nephritis (25). Previous reports demonstrated that CXCR3^+^ cells are recruited into inflamed kidneys in lupus prone mice and human lupus patients (26, 27). We showed that globally reducing FLI-1 levels in a lupus prone mouse strain reduced renal *Cxcl9* and *Cxcl10* gene expression and renal-infiltrating CXCR3+ T cells, and that FLI-1 can upregulate *Cxcr3* promoter activity in T cells (27). However, the underlying mechanisms by which FLI-1 modulates CXCL10 or CXCR3 expression remain unknown.

Our present study reports for the first time that FLI-1 impacts CXCL10 protein expression in kidneys of lupus mice and inflammatory cells. Although FLI-1 could directly bind to the *Cxcl10* promoter, it did not independently drive transcription *in vitro*. These results suggest FLI-1 may regulate *Cxcl10* through an indirect mechanism. Camptothecin (CPT), a clinically used chemotherapeutic drug, inhibited Fli-1 expression and reduced CXCL10 secretion in human renal endothelial cells. Fli-1 also drove CXCR3 promoter activity in T cells, which required the -89 to +200 region of the promoter and the DNA-binding domain of FLI-1. Our results also suggest that although the DNA-binding domain of FLI-1 is necessary for upregulating *hCXCR3* promoter activity, interaction with co-activators also may play a key role. Together, these results demonstrate a novel role for FLI-1 in modulating the CXCL10-CXCR3 inflammatory axis in lupus that may involve direct and indirect mechanisms of transcriptional regulation. Targeting signaling pathways or using drugs to reduce FLI-1 levels could be further explored as potential novel therapeutic approaches for LN.

## 2 Materials and methods

### 2.1 Mice

Wild-type MRL/*lpr* (Fli-1^+/+^) mice were purchased from the Jackson Laboratory (Bar Harbor, ME) and Fli-1^+/−^ littermates used in this study were bred from a colony maintained at our animal facility at Medical University of South Carolina. Generation of MRL/*lpr* Fli-1^+/−^ mice was described previously (14). Mice were housed in a pathogen-free environment and all procedures complied with the standards for care and use of animal subjects as stated in the *Guide for the Care and Use of Laboratory Animals* (Institute of Laboratory Resources, National Academy of Sciences, Bethesda, MD). The protocol for all animal studies was approved by the Institutional Animal Care and Use Committee at the Medical University of South Carolina. Kidneys were harvested from WT (Fli-1^+/+^) or Fli-1^+/−^ MRL/*lpr* mice at the age of 22 weeks, minced and lysed in RIPA lysis buffer. After lysis, the supernatant was collected. Total protein concentration was detected by BCA kit (Thermo Fisher Scientific, Waltham, MA) and CXCL10, CXCL9, and CXCL11 chemokine expression levels were measured by ELISA.

### 2.2 ELISA

CXCL10, CXCL9, and CXCL11 protein concentrations in the supernatants collected from the kidneys of MRL/*lpr* mice, human umbilical vein endothelial cells (HUVECs), MS1 cells, and human renal glomerular endothelial cells (HRGECs) were measured by mouse or human CXCL10, CXCL9 and CXCL11 ELISA kits (R&D Systems Inc., Minneapolis, MN) according to the manufacturer’s instructions.

### 2.3 Cells

The primary human umbilical vein endothelial cells (HUVECs) and primary human renal glomerular endothelial cells (HRGECs) were purchased from ScienCell Research Laboratories (Carlsbad, CA) and maintained in endothelial cell medium (Sciencell Research Laboratories, Carlsbad, CA) according to the manufacturer’s instructions. The murine endothelial MS1 cells and human Jurkat T cells were purchased from the American Type Culture Collection (ATCC). MS1 cells were cultured in Dulbecco’s Modified Eagle’s Medium (DMEM) (Mediatech Inc., Manassas, VA) with 10% fetal bovine serum and 1% penicillin/streptomycin. Jurkat T cells were cultured in RPMI 1640 with L-glutamine medium [Thermo Fisher Scientific (27)], 10 mM HEPES, 10% fetal bovine serum, and 1% penicillin/streptomycin. All cells were maintained at 37°C with 5% CO_2_.

### 2.4 Cell transfection and stimulation

HUVECs, HRGECs and MS1 cells were transfected with negative control or Fli-1 specific siRNA (Integrated DNA Technologies, Coralville, IA) for 24 hours and further stimulated with LPS (Sigma-Aldrich, St. Louis, MO; 0.025, 0.5, 1, 5, 10 µg/ml for MS1 cells, 0.1 µg/ml for HUVECs and HRGECs) or TNFα (10 ng/ml, Sigma-Aldrich, St. Louis, MO) for another 6 or 24 hours. Lipofectamine 2000 (Invitrogen, Waltham, MA) and serum-free Opti-MEM (Thermo Fisher Scientific, Waltham, MA) were used for the transfection. In addition, HRGECs were treated with Camptothecin (CPT, 0.25 µM, Selleckchem) for 24 hours and further stimulated with interferon α (IFNα, 100 ng/ml, Biolegend, San Diego, CA) or interferon γ (IFNγ, 100 ng/ml, Biolegend, San Diego, CA); for another 1, 4 or 24 hours. The cell supernatants were collected at different time points after stimulation.

Jurkat T cells were transfected with a pSG promoter/reporter (P/R) vector containing the human *CXCR3* (*hCXCR3*) proximal promoter (-435 to +284) driving Renilla luciferase (27). *hCXCR3* deletion constructs (-89/+284, +101/+284, and +200/+282) were generated by restriction digests on the 5’ end of the pSG *hCXCR3* -435/+284 vector to remove sequences followed by re-ligation. All deletion constructs were confirmed through direct sequencing. The P/R constructs were co-transfected into Jurkat T cells with a pcDNA vector expressing wild-type FLI-1 (pcDNA Fli-1) described previously (27), or a pSG vector expressing wild-type FLI-1 (pSG Fli-1), DNA binding mutant (pSG Fli-1 DBM), C-terminal transactivation domain truncation (pSG Fli-1 ΔCTA), acetylation K380 mutant (pSG Fli-1 Acet), or phosphorylation T312 mutant (pSG Fli-1 Phos) described previously (28, 29) using FuGENE (Promega, Madison, WI) according to manufacturer directions. Briefly, Jurkat cells were plated in 6-well plates and transfected with 0.5 μg *hCXCR3* P/R vector and the amount of Fli-1 expression vector indicated in figures legends. pGL3 Control (Promega, Madison, WI) expressing firefly luciferase at 0.1 μg was co-transfected and used to normalize for transfection efficiency. Transfected cells were incubated at 37°C, 5% CO_2_ for four hours, stimulated by addition of 10 ng/ml PMA and 100 ng/ml ionomycin, and incubated for another 16 hrs. Molar amount of transfected DNA was kept constant across wells by the addition of empty pSG or pcDNA expression vectors as appropriate. Renilla and firefly luciferase levels were measured by the Dual luciferase assay kit (Promega) as described previously (30). Transfections were performed in duplicate within an experiment and each experiment was performed twice with similar statistically significant differences observed across experiments. Representative results are presented. Significant differences were determined as described.

### 2.5 Western blotting

Cells were lysed in Radioimmunoprecipitation assay (RIPA) lysis buffer. After lysis, the supernatant was collected. Equal amounts of protein (20 µg-40 µg) were run for 1.5 hours at 130V on a 4–20% Criterion TGX Stain-Free Protein Gel (Bio-Rad, Hercules, CA) and electrotransferred to a PVDF membrane by Iblot2 transfer stacks (Invitrogen, Waltham, MA). Transferred proteins were probed with both a Fli-1 polyclonal antibody described previously (20) and an antibody to β-actin (Cell Signaling, Beverly, MA). The results were visualized using the Odyssey Imaging System (LI-COR, Lincoln, NE).

### 2.6 Chromatin immunoprecipitation sssay

The CXCL10 promoter was screened for putative Ets-1 DNA binding sites (EBSs) through visual inspection and utilizing the MatInspector software tool (Genomatix, Ann Arbor, MI) (31). Fourteen primer pairs were designed to cover the 46 putative EBSs identified (**Table 1**). ChIP assay was performed using an anti-Fli-1 rabbit polyclonal antibody or normal rabbit IgG control using EpiTect ChIP OneDay Kit (Qiagen, Germantown, MD) as described (32). Briefly, chromatin was isolated from cross-linked MS1 cells and immunoprecipitated (IP) by antibodies specific for Fli-1 or normal rabbit IgG. After IP, the DNA was purified and amplified by PCR according to the manufacturer’s instructions (Qiagen, Germantown, MD). Fold change was calculated using the comparative Ct method 2^−(ΔΔCT)^ where delta Ct was the difference between IgG and Fli-1 Ct values.

**Table 1:** Primers used in ChIP assay of CXCL10 promoter.

Primer Name	Forward Primer	Reverse Primer	Position from TSS	Amplicon Length (bp)
ChiP1	5’-GAG TCA TCT CCA AAG TCA G- 3’	5’-CAC TTG GGT TCA TGG TG-3’	-2082 to -1922	160
ChIP2	5’-GAA ACT TAC CTC ACT CG -3’	5’-CTG ACT TTG GAG ATG ACT C-3’	-1941 to 1788	153
ChIP3	5’-GAA CCT GAC TTA GAT ATC- 3’	5’-CCT CTT GTG CTC CTT TTA-3’	-1830 to -1660	170
ChIP4	5’-CTG CTC TAA CTG TTC AC -3’	5’-GAT ATC TAA GTC AGG TTC-3’	-1677 to -1501	176
ChIP5	5’-CTG TAA CCA CAC ACT CAC A- 3’	5’-GTG AAC AGT TAG AGC AG- 3’	-1518 to -1367	151
ChIP6	5’-GTT TTG AAC CGG TAC AC -3’	5’-CTT CTT TGT GAG TGT GTG G-3’	-1391 to -1184	207
ChIP7	5’ -CTT AGC TCT GTT CTA GTC- 3’	5’-GTG TAC CGG TTC AAA AC-3’	-1200 to -1027	173
ChIP8	5’-CTA TTC TGC AGA AGC AG-3’	5’-GAC TAG AAC AGA GCT AAG-3’	-1027 to -858	169
ChIP9	5’-CTG AGA ACT TGT ACA ATAAC-3’	5’-TGC TTC TGC AGA ATA GAC-3’	-873 to -733	140
ChIP10	5’-CTC TTG ACT AAC AGA TGC-3’	5’ -GTT ATT GTA CAA GTT CTC AG-3’	-753 to -602	151
ChIP11	5’-AAG CAG ACA CAG GCA AGT-3’	5’- CCT TAC TGA CGA GAA AAG-3’	-664 to -471	193
ChIP12	5’-CTG TTC TAG TCA AAG GG- 3’	5’- GCT GAA GTT ATC TTT GAT G-3’	-469 to -294	175
ChIP13	5’-CTT TTA TTT CAG TCA TTT GAC-3’	5’- GCA CTG AAT TAT AGC AGA- 3’	-285 to -129	156
ChIP14	5’-GAA TTC CGA CGT CTA CCT-3’	5’ -GTT AAT GTC AAA TGA CTG AA-3’	-156 to -1	155

Primers are listed based on their distance from the transcription start site (TSS).

### 2.7 DNA transfection

The mouse embryonic fibroblast NIH3T3 cell line was grown in DMEM (Mediatech, Manassas, VA) with 10% FBS and 1% penicillin/streptomycin. The NIH3T3 cell line was used for all transient transfection experiments because it is a commonly used cell line for DNA transfection, and the expression level of Fli-1 in these cells is undetectable. Twenty four hours prior to DNA transfection the NIH3T3 cells were seeded at a concentration of 4 × 10^5^ cells in 6 well plates. Cells were transfected using the FuGENE 6 transfection reagent (Promega, Madison, WI) following the manufacturer’s instructions. Reporter constructs were transfected into the cells at a concentration of 2 μg (pGL3/basic or pGL3/CXCL10) for all experiments. Increasing concentrations of the pcDNA/Fli-1 expression construct were used for the dose response study (0.05 μg, 0.1 μg, 0.2 μg, 0.25 μg, 0.5 μg, 1 μg and 2µg). Transfection of pcDNA/NF-κB p65 expression construct was used as positive control for driving *Cxcl10* promoter activity. In all experiments empty expression vectors were used to ensure that an equivalent amount of total DNA was transfected into the cells. The Renilla luciferase construct pRL/TK (Promega) was co-transfected into the cells at a concentration of 200 ng to control for transfection efficiency. The NIH3T3 cells were harvested 48 h after the transfection experiment was completed. To measure the luciferase activity for the transient transfection experiments the Dual-luciferase reporter assay system (Promega) was employed. The Luminoskan Ascent Microplate Luminometer (Thermo Fisher Scientific) was used to detect luminescence. All experiments were normalized using the Renilla luciferase expression. Fold activation is reported as the mean + standard error compared to the empty pGL3/basic vector.

### 2.8 Statistics

The data are expressed as the means ± SEM or SD as indicated in figure legends. GraphPad Prism 7 software (GraphPad Software, San Diego, CA) was used for statistical analysis. Two-group Student t tests (paired or unpaired as appropriate) were applied. Differences between the means of multiple groups were compared by the one-way analysis of variance (ANOVA), followed by a Tukey multiple comparisons test, and adjusted p-values presented. A value of *p*<0.05 was considered statistically significant.

## 3 Results

### 3.1 Renal CXCL10 and CXCL9 concentrations are significantly reduced in Fli-1^+/−^ MRL/*lpr* mice

**Figure 1:**
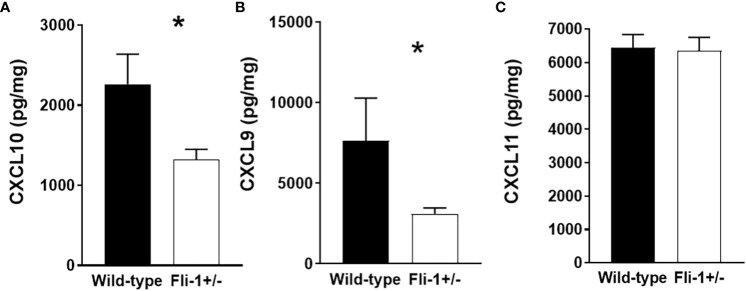
Decreased expression of CXCL10 and CXCL9 in kidneys from Fli-1^+/-^ MRL/*lpr* mice compared to wild-type littermates. The total protein was extracted and prepared from kidneys of Fli-1^+/-^ MRL/*lpr* mice and wild-type littermates at the age of 22 weeks (n = 12-13 in each group). Expressions of CXCL10 **(A)**, CXCL9 **(B)**, and CXCL11 **(C)** in kidneys were measured by ELISA. Data were shown as mean ± SEM. * indicates *p* < 0.05.

Genetically reducing the expression of Fli-1 in two lupus mouse models significantly reduced renal damage with profoundly decreased infiltrating inflammatory cells, and prolonged survival (14, 15, 20, 33). We previously showed that the gene expression of *Cxcl9* and *Cxcl10* was reduced in the kidney of the MRL/*lpr* Fli-1^+/-^ mice (27). To assess if CXCL9, or CXCL10 protein expression are also reduced in these mice, we measured the concentrations of these chemokines in the kidney of Fli-1^+/−^ and wild-type littermates at the age of 22 weeks. Renal levels of CXCL10 (**Figure 1A**) and CXCL9 (**Figure 1B**), but not CXCL11 (**Figure 1C**), in Fli-1^+/−^ MRL/*lpr* mice were significantly lower compared to wild-type littermates. Thus, renal CXCL9 and CXCL10 are decreased at the transcript and protein levels in lupus prone MRL/*lpr* Fli-1^+/^ **^−^** ^-^ mice and suggests FLI-1 modulates CXCL10, CXCL9 expression in the kidneys during lupus nephritis.

### 3.2 Reduced *Fli-1* expression results in decreased secretion of CXCL10 in human and mouse endothelial cells

**Figure 2:**
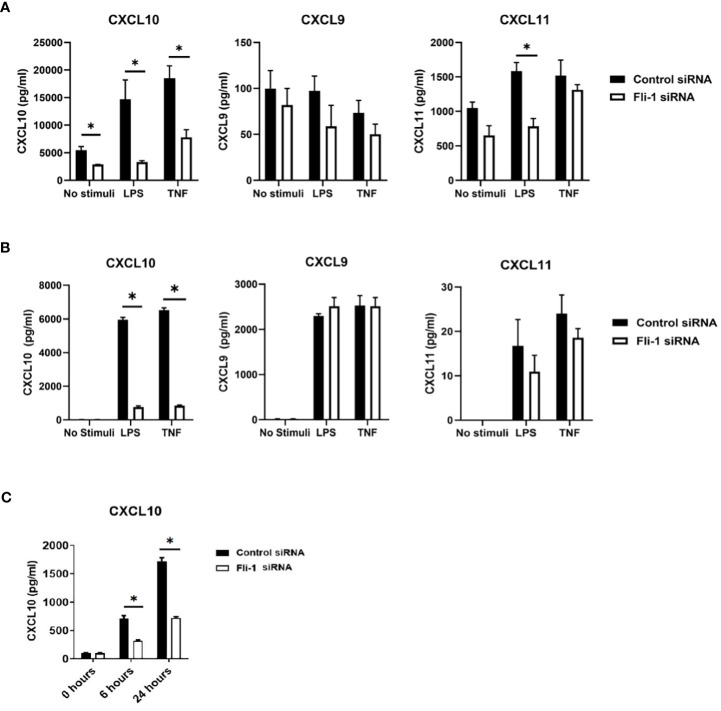
Inhibition of Fli-1 expression decreased production of CXCL10 in human umbilical vein endothelial cells (HUVECs), human renal glomerular endothelial cells (HRGECs), and mouse endothelial MS1 cells. HUVECs **(A)**, or HRGECS **(B)** were transfected with Fli-1 specific or negative control siRNA for 24 hours and further stimulated with LPS (0.2 µg/ml) or TNFα (10 ng/ml) 24 hrs. Secretion of CXCL10, CXCL9, and CXCL11 in supernatant were measured by ELISA. N=3-4 in each group. The data were represented as mean ± SEM. * indicates *p* < 0.05. **(C)** MS1 endothelial cells were transfected with Fli-1 specific or negative control siRNA for 24 hours and stimulated with LPS (0.1 µg/ml) for another 6 or 24 hours. Secreted CXCL10, CXC9 and CXCL11 concentrations were measured by ELISA. CXCL9 and CXCL11 were undetectable. N=4 in each group. The data were represented as mean ± SEM. * indicates *p*< 0.05.

Kidney endothelial cells produce significant inflammatory cytokines and are implicated in nephritis development, and we demonstrated that FLI-1 is highly expressed in renal endothelial cells (20, 34). To assess if FLI-1 regulates the expression of CXCL9, CXCL10, or CXCL11, the primary human umbilical vein endothelial cells (HUVECs) were employed. HUVECs transfected with FLI-1 specific siRNA to knockdown FLI-1 expression produced significantly lower levels of CXCL10 compared to HUVECs transfected with control siRNA in response to LPS or TNF-α stimulation (**Figure 2A**). CXCL9 was not elevated by LPS or TNFα stimulation, and reducing FLI-1 expression had no significant effect on CXCL9 levels (**Figure 2A**). CXCL11 was significantly reduced in cells transfected with FLI-1 siRNA compared to cells transfected with control siRNA following LPS stimulation only. Reduction of FLI-1 protein in the cells transfected with FLI-1 siRNA was confirmed by immunoblotting (**Supplementary Figure S1A**).

To confirm the results above and demonstrate relevance to lupus nephritis, the effects of FLI-1 on the production of CXCL10 in primary human renal glomerular endothelial cells (HRGECs) were assessed. HRGECs transfected with FLI-1 specific siRNA had reduced FLI-1 protein levels (**Supplementary Figure S1B**). LPS and TNF-α induced CXCL10 production at 24 hours, which were significantly suppressed by knockdown of FLI-1 (**Figure 2B**). CXCL9 and CXCL11 were also induced by LPS and TNFα, but were not significantly impacted by knockdown of FLI-1 (**Figure 2B**). These results suggest FLI-1 modulates CXCL10, but not CXCL9 or CXCL11 in primary HRGECs.

To determine if FLI-1 also regulates CXCL10 expression in mouse endothelial cells, *Fli-1* specific siRNA or negative control siRNA was transfected into the murine endothelial cell line MS1. Reduced FLI-1 protein in the cells transfected with FLI-1 siRNA was confirmed by immunoblotting (**Supplementary Figure S1C**). LPS significantly elevated CXCL10 production in a dose-dependent manner (data not shown) in MS1 cells, and transfection with *Fli-1* siRNA significantly decreased production of CXCL10 compared to the cells transfected with negative control siRNA at 6 and 24 hours (**Figure 2C**). CXCL9 and CXCL11 production from MS1 cells was undetectable in response to LPS (data not shown). Together, these data demonstrate that FLI-1 modulates the production of CXCL10 in both human and mouse endothelial cells.

### 3.3 FLI-1 binds to the *Cxcl10* promoter

**Figure 3:**
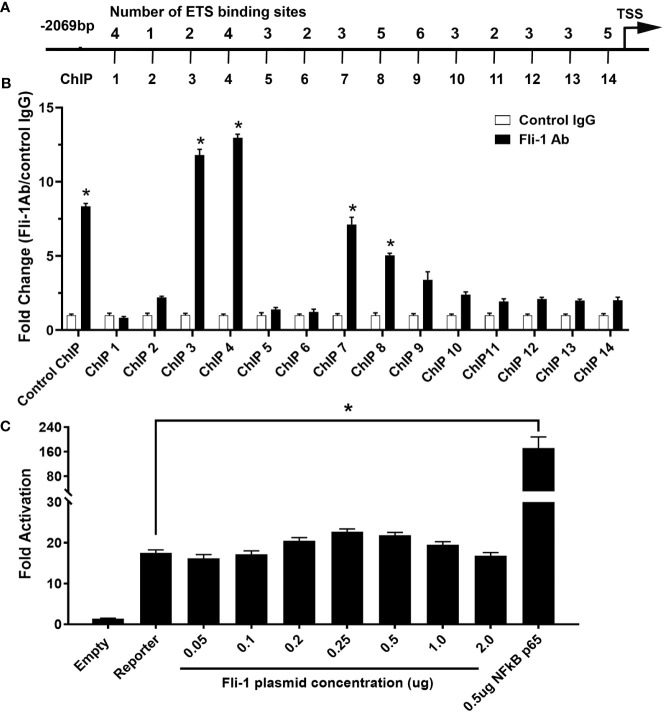
ChIP analysis of Fli-1 binding to the CXCL10 promoter, but Fli-1 failed to drive transcription from the CXCL10 promoter. **(A)** Putative Fli-1 binding sites within the murine CXCL10 promoter and the location of the primers used for the ChIP assay. The number of potential binding sites for each primer set is noted above. **(B)** FLI-1 binding in the ChIP assay was determined by enrichment of the indicated CXCL10 regions by IP with anti-Fli-1 antibody relative to enrichment of regions by IP with a control IgG antibody. A pair of primers for the MCP1 promoter previously shown to be enriched in a ChIP assay by anti-Fli-1 antibody was used as ChIP Control. * Indicates *p*<0.05. Data were represented as mean ± SEM from three independent experiments. **(C)** Human CXCL10 promoter cloned into the pGL3 vector, upstream of the luciferase reporter gene co-transfected with increasing amounts of a Fli-1 expression vector (0.05, 0.1, 0.2, 0.25, 0.5, 1 and 2 μg). Co-transfection of a NFkBp65 expression vector (0.5µg) was used as positive control. Values shown are fold activation over the empty vector control (mean + SEM for three replicate experiments; n = 9), * Indicates p<0.05.

To determine if FLI-1 directly regulates CXCL10 production by binding to its promoter, we identified 46 putative ETS binding sites on the mouse *Cxcl10* promoter and designed 14 primer pairs to cover all 46 sites (**Figure 3A** and **Table 1**). ChIP assays using these 14 primer pairs with MS1 cells were performed. Our previous study showed that FLI-1 regulates several chemokine genes through binding to their promoters, including MCP1 (32). Therefore, 1 pair of primers from the MCP1 promoter was used as a positive control for ChIP (ChIP Con, **Figure 3B**). Promoter regions ChIP3, ChIP4, ChIP7, and ChIP8 were significantly enriched more than 4-fold by FLI-1 specific antibodies compared to the IgG negative control. These results indicate that FLI-1 can bind directly to the *Cxcl10* promoter and likely modulates CXCL10 production by upregulating its transcription in LPS- and TNFα-stimulated endothelial cells.

### 3.5 FLI-1 failed to directly drive transcription from the human *CXCL10* promoter

Transient transfection assays were performed to determine if FLI-1 can regulate the human *CXCL10* gene. A pGL3/*hCXCL10* promoter/reporter construct was used to determine if FLI-1 can drive transcription from the *hCXCL10* promoter. The pGL3/*hCXCL10* construct co-transfected with increasing amounts of a FLI-1 expression vector into the NIH3T3 cell line. FLI-1 failed to drive transcription from the human *CXCL10* promoter even at high doses of the FLI-1 expression vector (**Figure 3C**). Co-transfection of a NF-κB p65 expression vector with the pGL3/hCXCL10 reporter construct was able to upregulate *hCXCL10* promoter activity as reported previously (**Figure 3C**) (35). FLI-1 also failed to drive transcription from the mouse *Cxcl10* promotor in the HUVECS (data not shown). These results indicate that FLI-1 may regulate the expression of *CXCL10* through an indirect or novel mechanism. Alternatvely, the cell lines used in these studies lacked an important co-activator necessary for FLI-1 to drive, or contained a corepressor that inhibited FLI-1 from driving, *CXCL10* expression.

### 3.6 CPT, a potential Fli-1 inhibitor, reduced CXCL10 production in human renal glomerular endothelial cells

**Figure 4:**
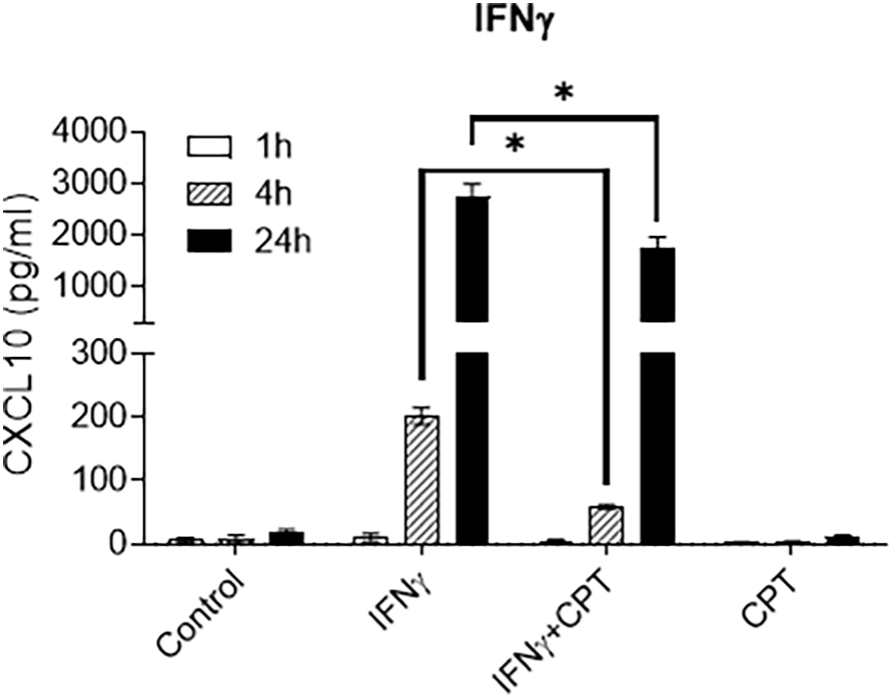
CPT inhibits Fli-1 expression and reduces CXCL10 production in HRGECs. HRGECs were treated with CPT at 0.25 µM or a DMSO vehicle control for 24 hours and CXCL10 concentrations in media from HRGECs were measured by ELISA at 1, 4 and 24 hours after stimulation with 100 ng/ml of IFN-γ N=4 in each group. ^*^ *p*<0.05.

Camptothecin (CPT) was identified as an effective Fli-1 inhibitor in a screen of known small molecule/compound libraries (36). Thus, CPT was tested to determine if it inhibits endogenous FLI-1 expression in HRGECs. HRGECs treated with CPT for 24 hours at 0.25 µM demonstrated that CPT inhibited FLI-1 protein expression (**Supplementary Figure 2**). Moreover, 0.25 µM CPT significantly reduced IFNγ-induced CXCL10 secretion in HRGECs at 4 and 24 hours (**Figure 4**).

### 3.7 The DNA-binding domain of FLI-1 is necessary for its regulation of CXCR3 promoter activity in T cells

**Figure 5:**
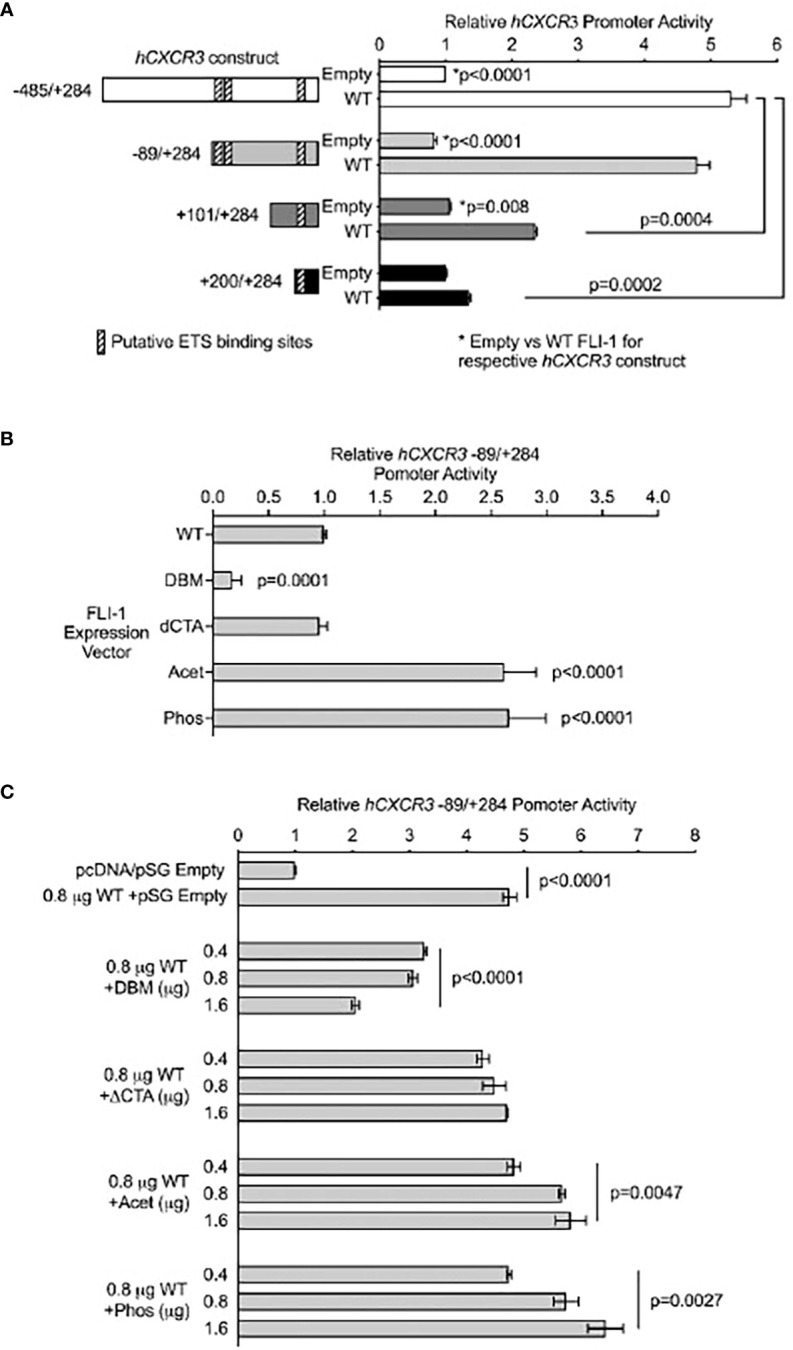
FLI-1 directly regulates promoter activity of the human CXCL10 receptor *hCXCR3*. Jurkat T cells were transiently transfected with *hCXCR3* promoter/reporter (P/R) constructs without or with FLI-1 expression vectors and stimulated by addition of PMA and ionomycin. **(A)** *hCXCR3* deletion P/R constructs indicated in the graph were co-transfected with 0.8 μg of the expression vector pcDNA without (Empty) or with wild-type FLI-1 (WT). Locations of putative FLI-1 binding sites in *hCXCR3* identified previously (27) are indicated as hatched boxes on the constructs depicted to the left of the graph. The full-length -485/+284 construct co-transfected with the Empty expression vector was set to 1 with all other values relative to its expression. *p-values on the graph compares transfection with WT to Empty for each construct and the other p-values on the graph compares the WT transfected deletion *hCXCR3* constructs to the WT transfected -485/+284 construct. **(B)** *hCXCR3* -89/+284 P/R vector was co-transfected with a pSG WT FLI-1 (WT) expression vector or the pSG FLI-1 mutant expression vectors indicated on the graph. DNA-binding mutant (DBM); deletion of the C-terminal transactivation domain (ΔCTA); acetylation mutant (Acet); phosphorylation mutant (Phos). Transfection with the pSG WT vector was set to 1 and expression with the FLI-1 mutants is relative to WT. *p*-values are comparison of each mutant to WT FLI-1. **(C)** *hCXCR3* -89/+284 P/R vector was co-transfected with pcDNA Empty (0.8 μg) +pSG Empty (1.6 μg), pcDNA WT FLI-1 (0.8 μg) +pSG Empty (1.6 μg), or pcDNA WT FLI-1 (0.8 μg) +increasing amounts (0.4, 0.8, 1.6 μg) of the pSG FLI-1 mutants indicated. All values are presented relative to expression of transfection with the Empty pcDNA and pSG vectors, which was set to 1. *p*-values on the graph include comparison on WT +pSG Empty to pcDNA/pSG Empty, and global *p*-values for WT +increasing amounts of each mutant compared to WT +pSG Empty. Transfections were performed in duplicate, and each transfection was performed twice with similar results. Data presented as relative promoter activity and are the average of means ±SD. *p*-values were calculated as described in materials and methods.

CXCL10 expression helps recruit cells expressing its receptor CXCR3. A previous study from our lab demonstrated that reducing FLI-1 levels in MRL/*lpr* mice significantly reduced the numbers of renal infiltrating CXCR3^+^ T cells and that FLI-1 regulates the promoter activity of the human *CXCR3* (*hCXCR3*) promoter in T cells (27). To further examine the mechanism by which FLI-1 regulates *hCXCR3* promoter activity, Jurkat T cells were co-transfected with a pcDNA vector expressing wild-type FLI-1 and either the full-length *hCXCR3* (-485/+284) or various *hCXCR3* 5’ deletion promoter/reporter (P/R) constructs (**Figure 5A**). Results indicate that most of the FLI-1 regulated *hCXCR3* promoter activity is located between -89 and +101 (**Figure 5A**). Our previous results also suggested that although the DNA binding region of FLI-1 was required for upregulating *hCXCR3* promoter activity, FLI-1 binding to three conserved putative ETS binding sites with high homology for FLI-1 was not detected in *in vitro* binding assays (27). Two of these three putative binding sites are located within the -89 to +100 region. The third site, and least homologous for FLI-1, is located between +200 and +284 region of the *hCXCR3* promoter.

To further determine whether FLI-1 regulates *hCXCR3* by directly binding and transactivating the promoter, Jurkat T cells were transfected with P/R construct *hCXCR3* -89/+284 containing all three putative FLI-1 binding sites and similar activity to the full-length -435/+284 construct along with expression vectors for wild-type (WT) or various mutants of FLI-1. As we demonstrated previously with the full-length -485/+284 promoter construct (27), the FLI-1 DNA binding mutant (DBM) does not upregulate -89/+284 *hCXCR3* promoter activity (**Figure 5B**). Interestingly, a FLI-1 mutant missing the C-terminal transactivation domain (ΔCTA) does not impact the ability of FLI-1 to upregulate hCXCR3 promoter activity while mutations preventing FLI-1 acetylation (Acet) or phosphorylation (Phos) upregulated the activity of the *hCXCR3* promoter better than wild-type FLI-1. We then used a competition assay to further assess the mechanism by which FLI-1 upregulates -89/+284 *hCXCR3* promoter activity. For this experiment, WT FLI-1 was co-transfected without or with increasing amounts of each FLI-1 mutant. The FLI-1 DBM significantly and dose-dependently inhibited, while the Acet and Phos FLI-1 mutants further increased, *hCXCR3* promoter activity stimulated with WT FLI-1 (**Figure 5C**). These results show that although FLI-1 requires its DNA binding domain to upregulate *hCXCR3*, it can block WT FLI-1 from activating the *hCXCR3* promoter. This suggests the FLI-1 DBM likely inhibits WT FLI-1 by interacting with and sequestering co-activators needed for FLI-1 to upregulate *hCXCR3* promoter activity. Furthermore, the acetylation and phosphorylation status appear to modulate FLI-1 activity in regulating *hCXCR3*.

## Discussion

Chemokines, such as CXCL10, play a critical role in the inflammatory/autoimmune response, particularly in recruiting inflammatory/immune cells expressing receptors for chemokines, such as CXCR3, to areas of disease or injury (24, 37, 38). A recent report demonstrated that CXCR3 deficiency decreases autoantibody production and inhibits aberrant activated T follicular helper cells and B cells in lupus mice (39). Elevated expression of CXCL10 is strongly correlated with the pathogenesis of clinical LN, neuropsychiatric lupus (NPSLE), and the disease activity of SLE (24, 25, 40). A majority of renal infiltrating T cells, a hallmark of nephritis in SLE patients, express CXCR3, the receptor for CXCL10 (26, 41). CXCR3^+^ T cells are mainly observed in the interstitial infiltrate located in periglomerular and perivascular regions of the kidney. In these regions, *CXCR3* mRNA expression correlated with *CXCL10* mRNA expression, CXCR3^+^ T cells co-localized with CXCL10-producing cells, and both expression levels and numbers of cells expressing CXCR3 correlated with clinical markers of disease (26, 41). Loss of CXCR3 in the MRL/*lpr* lupus strain results in significantly reduced renal T cell infiltration and improved disease (42). Our previous studies demonstrated that MRL/*lpr* lupus mice with reduced FLI-1 levels exhibited decreased renal *Cxcl9* and *Cxcl10* mRNA levels and reduced numbers of CXCR3^+^ T cells infiltrating the kidneys (27). Our present study confirmed that renal CXCL9 and CXCL10 expression at the protein level are significantly decreased in Fli-1^+/−^ MRL/*lpr* mice. We also demonstrated that FLI-1 impacts the expression of CXCL10 in human and mouse endothelial cells, and CXCR3, the receptor for CXCL10, in human T cells. Together, the combined results strongly suggest an important role for FLI-1 in modulating the CXCL10-CXCR3 axis and thus, in promoting nephritis, further supporting FLI-1 as potential therapeutic target.

FLI-1 impacts glomerulonephritis development by regulating inflammatory chemokines in endothelial cells, as well as inflammatory cell infiltration in the kidneys during lupus (10, 14, 18, 27, 32, 33, 43, 44). Here we showed that FLI-1 knockdown reduced production of CXCL10 in all endothelial cells tested and under all stimulatory conditions while production of CXCL9 and CXCL11 was not impacted or was impacted in only certain cells/conditions. Hence, the impact of FLI-1 on CXCL10 expression was consistent between the mouse kidney tissue and mouse or human endothelial cells. *CXCL10* is regulated by nuclear factor kappa B (NF kappa B) protein, cRel, through direct binding to the promoter in mouse embryo fibroblasts (45). Signaling pathways p38, JNK, ERK and Akt also were reported to regulate the transcription of *CXCL10* (38), highlighting the complex mechanisms involved in the transcriptional regulation of *CXCL10*. Epithelial cells were found to increase their expression of CXCL10 in response to IFN stimulation (46). However, it should be noted that Fli-1 is not detectable in epithelial cells (data not shown), and therefore, we have not yet investigated the impact of Fli-1 on CXCL10 expression in these cells. While our results show that FLI-1 can directly bind to the *CXCL10* promoter, FLI-1 did not drive transcription from the human or mouse CXCL10 promoters. There are several potential explanations to interpret this result. First, a co-factor not expressed by NIH3T3 cells may be required for Fli-1 to drive transcription from the CXCL10 promoter, or these cells express a corepressor that inhibits Fli-1 activation of the CXCL10 promoter. We attempted to address this by transfecting the Fli-1 expression vector into HUVEC cells, along with the full-length CXCL10 promoter. However, Fli-1 also failed to drive transcription in HUVEC cells. Another possible explanation is that the binding of Fli-1 to the CXCL10 promoter alters the chromatin structure, which could either prevent or enhance the binding of other factors involved in regulating CXCL10. In fact, a recent report concluded that FLI-1 modulates the expression of many genes by influencing chromatin accessibility (47). Thus, FLI-1 may be indirectly regulating *CXCL10* expression by altering the chromatin structure of the gene. Understanding the mechanisms by which CXCL10-CXCR3 axis is regulated could assist in the generation of therapeutic interventions for human diseases.

We also demonstrated previously that in T cells, FLI-1 drives transcription from the promoter of CXCR3, the receptor for CXCL10 (27). In this study, we further determined that maximal upregulation of CXCR3 promoter activity by FLI-1 requires the -89 to +100 region of the promoter, which contains two putative ETS binding sites. Additionally, we demonstrated that although a DNA-binding mutant of FLI-1 could not upregulate CXCR3 promoter activity, it significantly inhibited activation of the CXCR3 promoter by wild-type FLI-1, likely by sequestering a required co-factor. The FLI-1 construct lacking the C-terminal transactivation (CTA) domain retained its ability to activate the CXCR3 promoter. Together, these results suggest FLI-1 activates the CXCR3 promoter by binding to the promoter and interacting with transcriptional co-activators outside of its DNA-binding or CTA domains. We were unsuccessful in demonstrating direct binding of FLI-1 to the *CXCR3* promoter *in vitro* (27); however, the conditions may not have been ideal for FLI-1 binding *in vitro.* Future studies aimed at assessing *in vivo* binding will likely be more informative. In addition, based on our results, the phosphorylation and acetylation status appears to regulate FLI-1 function in transcriptional activation of the CXCR3 promoter. Thus, post-translational modifications of FLI-1 also may play a role in regulating CXCR3, which was previously demonstrated for its regulation of the G-CSF or COL1A2 promoters (21, 28, 48).

Certain regimens incorporating cytotoxic drugs, especially cyclophosphamide, provide a significant therapeutic advantage over corticosteroids alone in the management of lupus disease (49). Cytotoxic drugs frequently have adverse effects and vary from person to person during the lupus treatment process. Since reducing FLI-1 decreases expression of CXCL10, FLI-1 may be an alternative drug target for lupus, an ongoing focus in our lab. Cytotoxic drug CPT is a FLI-1 inhibitor screened from known small molecule/compound libraries (36). CPT has been widely used in cancer therapy since the late 20th century (50). Herein we demonstrated that CPT significantly inhibited FLI-1 protein levels in a dose-dependent manner and reduced IFNγ-induced CXCL10 secretion in human renal endothelial cells. Indeed, low dose CPT markedly ameliorated lupus nephritis in (NZB × NZW)F1 mice (51). The role of CXCL10/CXCR3 in anti-GBM nephritis has been well established (52). Future studies are aimed at investigating the impact of CPT on nephritis by inhibiting Fli-1 modulation of the CXCL10/CXCR3 axis.

In summary, our studies support FLI-1 as a novel transcription factor modulating gene expression of inflammatory chemokine *CXCL10* in endothelial cells and its receptor *CXCR3* in T cells. Considering the emerging evidence that the CXCL10-CXCR3 axis plays a critical role in lupus and other inflammatory/autoimmune diseases, our results provide additional insight in defining the mechanisms by which FLI-1 modulates inflammation and disease development. Inhibition of FLI-1 may serve as a therapeutic approach for reducing inflammatory diseases by preventing upregulation of the CXCL10-CXCR3 axis in addition to multiple other cytokines, all of which contribute to renal inflammation and injury in LN (10, 18, 20, 32). Thus, we provide additional evidence that FLI-1 may serve as a global key regulator of inflammation.

## Data availability statement

The raw data supporting the conclusions of this article will be made available by the authors, without undue reservation.

## Ethics statement

Ethical approval was not required for the studies on humans in accordance with the local legislation and institutional requirements because only commercially available established cell lines were used. The animal study was approved by The Institutional Animal Care and Use Committee/Medical University of South Carolina. The study was conducted in accordance with the local legislation and institutional requirements.

## Author contributions

Study conception and design: XZ, TN: Acquisition of data: XW, XZ, TN, ML, TSC, KS, SS, Analysis and interpretation of data: XW, XZ, TN, ML, TSC, KS, SS. All authors contributed to the article and approved the submitted version.
