# Supplementary material for: Role of the transcription factor Fli-1 on the CXCL10/CXCR3 Axis*
Source: Front Immunol. 2023 Sep 15;14:1219279. doi: 10.3389/fimmu.2023.1219279 (PMC10543418; doi:10.3389/fimmu.2023.1219279)
Supplement: Supplementary file 1 [file DataSheet_1.docx]

**Support supplemental materials.**

Figure 1. Fli-1 protein was reduced in the cells transfected with Fli-1 siRNA.


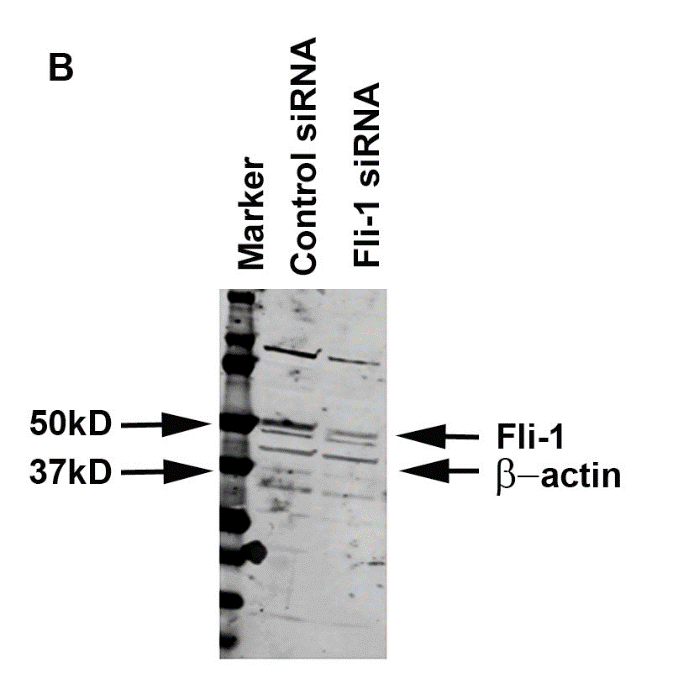


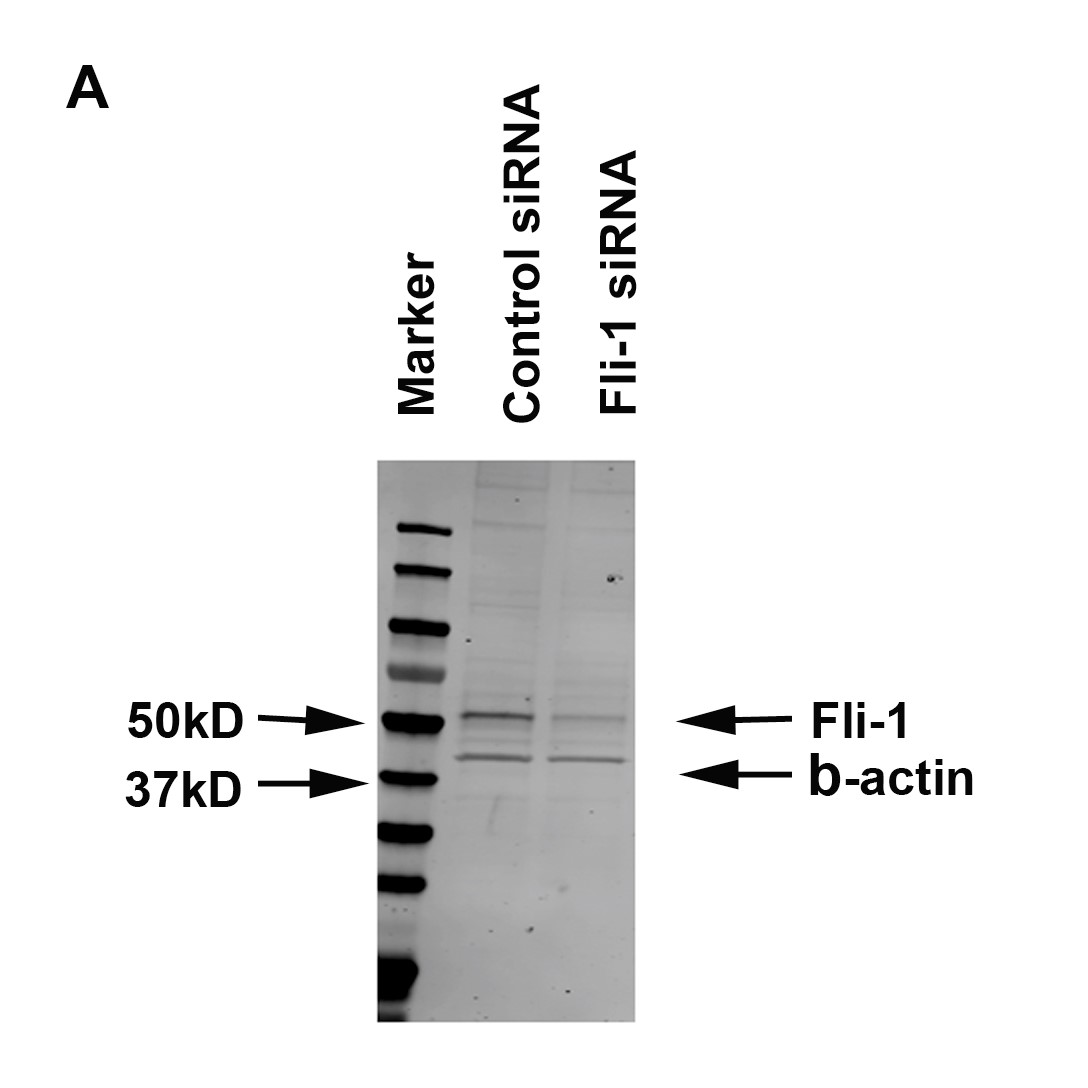


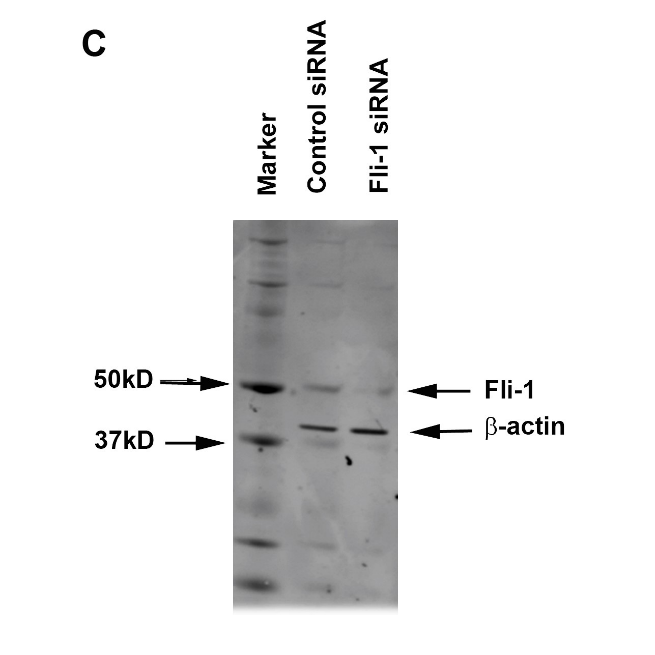


Human umbilical vein endothelial cells (HUVECS) (A), human renal glomerular endothelial cells (HRGECs) (B) or MS1 cells (C) were transfected with Fli-1 specific or negative control siRNA for 24 hours and Fli-1 protein levels were determined by Western Blot.

Figure 2. FLI-1 protein was reduced in the HRGECs after treatment with camptothecin.


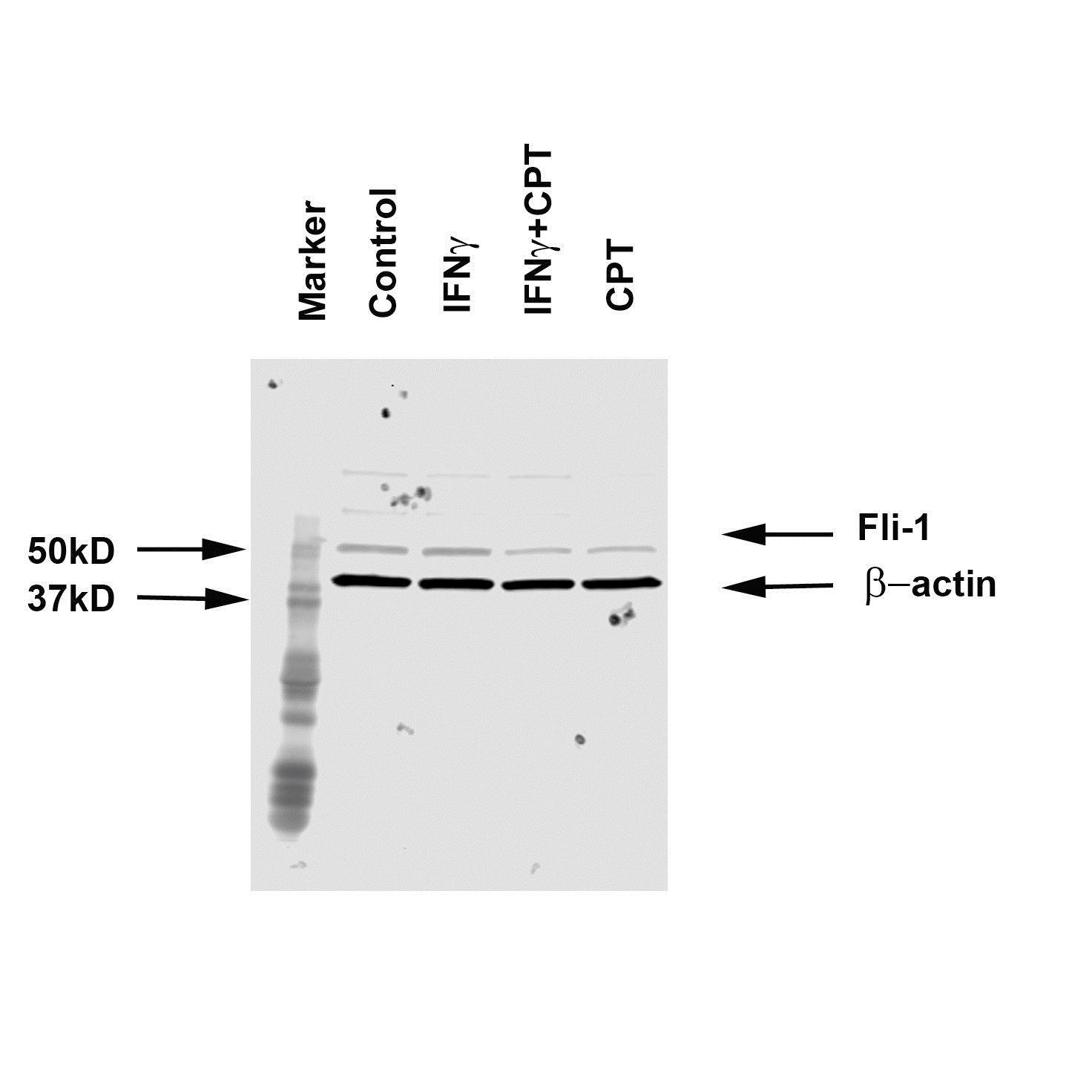


HRGECs were treated with camptothecin at 0.25 µM or a DMSO vehicle control for 24 hours and then lysed for immunoblotting to detect FLI-1 protein.
